# A rare case of Cytomegalovirus enteritis in a 65 year old immunocompetent Caucasian male following cardiac surgery

**DOI:** 10.1186/1749-8090-10-S1-A30

**Published:** 2015-12-16

**Authors:** Simon Mbarushimana, Asif Farooqui, Harry Parissis, Aaron Ervine

**Affiliations:** 1 Department of General Surgery and Anaesthetics, Altnagelvin Area Hospital, Glenshane Road, Derry, Northern Ireland, BT47 6SB, United Kingdom; 2 Department of Cardiothoracic Surgery, Royal Victoria Hospital, Belfast, Northern Ireland, BT12 6BA, United Kingdom; 3 Department of Histopathology, Royal Victoria Hospital, Belfast, Northern Ireland, BT12 6BA, United Kingdom

## Background/Introduction

Cytomegalovirus (CMV) enteritis post coronary artery bypass grafting (CABG) has been previously reported but never in an immunocompetent patient not on steroid therapy preoperatively.

## Aims/Objectives

CMV enteritis must be considered and actively sought in immunocompetent patients who develop GI complications post CABG

## Method

A MEDLINE literature search and manual review of the bibliographies of relevant papers failed to identify any other histologically proven case of post CABG CMV enteritis (Figure 1) in a patient who was immunocompetent, not on steroid therapy preoperatively and whose revascularization was done on a beating heart

## Results

A 65 year old immunocompetent gentleman presented with signs/symptoms of crescendo angina and an elevated troponin (297ng/L). Angiography showed triple vessel disease and severe left ventricular dysfunction. Cardiac Magnetic Resonance Imaging demonstrated some reversibility but a low ejection fraction of 20%. He underwent urgent CABG, and as per protocol was admitted to the cardiac intensive care unit (CICU).

Postoperatively, he developed multiple complications including lower and upper gastrointestinal (GI) bleeding, acute abdomen and dialysis resistant lactate acidosis. Due to his abdominal symptoms/signs, he underwent a laparotomy, resection of 82 cm of necrotic perforated small bowel and fashioning of an end ileostomy.

Histology proved the aetiology of his abdominal symptoms/signs was CMV enteritis.

## Discussion/Conclusion

1. Consider CMV enteritis in immunocompetent patients who have complicated cardiac ICU stay and signs and symptoms such as abdominal pain, watery or bloody diarrhoea, bleeding, obstruction, toxic megacolon, perforation and fistula formation.

2. Have a high index of suspicion for CMV enteritis in patients on steroids pre-operatively who have complicated ICU recovery and shows the above signs and symptoms.

3. As mortality for CMV enteritis can be as high as 80%, early diagnosis and treatment are crucial.

## Consent

Written informed consent was obtained from the patient for publication of this abstract and any accompanying images. A copy of the written consent is available for review by the Editor of this journal.

